# Resveratrol Increases Hepatic *SHBG* Expression through Human Constitutive Androstane Receptor: a new Contribution to the French Paradox

**DOI:** 10.1038/s41598-017-12509-x

**Published:** 2017-09-25

**Authors:** Cristina Saez-Lopez, Laura Brianso-Llort, J. Torres-Torronteras, Rafael Simó, Geoffrey L. Hammond, David M. Selva

**Affiliations:** 1 Diabetes and Metabolism Research Unit, Vall Hebron Institut de Recerca (VHIR). Universitat Autònoma de Barcelona and Biomedical Network Research Centre on Diabetes and Metabolic Diseases (CIBERDEM, ISCIII), Barcelona, Spain; 2 Research Group on Neuromuscular and Mitochondrial Diseases, Vall Hebron Institut de Recerca (VHIR). Universitat Autònoma de Barcelona and Biomedical Network Research Centre on Rare Diseases (CIBERER, ISCIII), Barcelona, Spain; 3 Cellular & Physiological Sciences, University of British Columbia, Vancouver, Canada

## Abstract

Sex hormone-binding globulin (SHBG) carries sex steroids in blood regulating their bioavailability. Red wine consumption increases plasma SHBG levels, and we have discovered that resveratrol, a polyphenol enriched in red wine, acts specifically through the human constitutive androstane receptor (CAR), a drug/xenobiotic detoxification gene regulator, to increase hepatic SHBG production. Chromatin immunoprecipitation and luciferase reporter gene assays show that human CAR binds to a typical direct repeat 1 nuclear hormone receptor-binding element in the human *SHBG* proximal promoter. Resveratrol also increased hepatic SHBG production in humanized *SHBG*/*CAR* transgenic mice. Moreover, *SHBG* expression correlated significantly with CAR mRNA levels in human liver biopsies. We conclude that the beneficial effects of red wine on the metabolic syndrome and it associated co-morbidities, including cardiovascular disease and type 2 diabetes, may be mediated in part by resveratrol acting via CAR to increase plasma SHBG levels.

## Introduction

Sex hormone-binding globulin (SHBG) is produced and secreted by the human liver into the blood, where it binds androgens and estrogens with high affinity, regulating their bioavailability^1^. Low plasma SHBG levels are associated with obesity and the metabolic syndrome predicting risk for type 2 diabetes^2–5^ and cardiovascular disease^4,6,7^. Studies of human *SHBG* regulation have revealed *cis*-acting elements in the human *SHBG* proximal promoter that interact with members of the nuclear hormone receptor (NHR) family, including HNF4α and COUP-TF that compete for an imperfect NHR DR-1 (direct repeat-1) element located about 20 bp upstream of the transcription start site, and act as the main stimulators and repressors of *SHBG* expression in liver cells, respectively^8^. In addition, PPARγ interacts with a consensus DR-1 element located about 50 bp further upstream, which also binds HNF4α and COUP-TF^8^, and PPARγ binding at this position represses *SHBG* transcription^9^.

Olive oil and red wine are important components of the Mediterranean diet^10^ that are associated with a decreased risk of cardiovascular disease^11–14^. We have shown that olive oil consumption is associated with elevated serum SHBG levels and that PPARγ downregulation induced by oleoyl-CoA is an underlying mediator of this effect^15^. Numerous epidemiological studies indicate that cardiovascular risk is decreased by moderate wine consumption, providing an explanation for the “French Paradox”^16–19^. The beneficial cardiovascular effects of wine consumption have been attributed to components other than alcohol^20–22^, which are generally referred to as congeners of alcoholic beverages. These include the polyphenols, and red wine contains between 5 and 20-fold higher concentrations of polyphenols than does white wine^23,24^. Among these polyphenols, resveratrol (3,4′,5-trihydroxystilbene) is a potent antioxidant that is considered protective against cardiovascular disease, cancer, age-related deterioration and the deleterious consequences of high-fat diets^20,22–28^. Regarding the liver protective effects of resveratrol against high fat diets, several mechanisms have been involved such as the increase of carnitine palmitoyl transferase-Iα and the inhibition of acyl-coenzyme A carboxylase, fatty acid synthase, glucose-6-phosphate dehydrogenase, and phosphatidate phosphohydrolase^28–30^. Several biochemical pathways may contribute to the cardiovascular protection afforded by resveratrol^16,31–33^, including its ability to activate the constitutive androstane receptor (CAR, NR1I2) that belongs to the NR1I subfamily of NHRs^34^. In mammals, CAR plays a crucial role in regulating drug metabolism, and is a key drug/xenobiotic-sensitive transcriptional regulator^35–38^. Human CAR activation induces the expression of several CYP enzymes^39,40^ and genes encoding phase II enzymes like the uridine diphosphate glucuronosyltransferase (UGT) isoforms^41–43^. To regulate gene expression, CAR binds a functional enhancer, namely the phenobarbital-responsive enhancer module^44,45^. Human CAR can also compete with HNF-4α for binding to DR-1 elements in the *CYP7A1*, *CYP8B1*, and *PEPCK* promoters^46^. The affinity of different ligands for CAR also varies between species^47^; for instance human CAR is strongly activated by CITCO, while murine CAR is not^48^ and is more sensitive to TCPOBOP than human CAR^49,50^.

Experiments in which the addition of red wine to culture medium increased SHBG production by human HepG2 liver cells led us to discover that resveratrol was responsible for this effect through a specific interaction with human CAR binding to a DR-1 element in the *SHBG* promoter. Interestingly, we also found that SHBG mRNA levels correlated positively with CAR in human liver biopsies. These findings provide mechanistic insight into the beneficial health effects of resveratrol and how it might contribute to reducing risk for the metabolic syndrome and its associated comorbidities.

## Results

### Resveratrol increases SHBG production independently of alterations in HNF-4α and PPARγ levels in HepG2 cells

**Figure 1:**
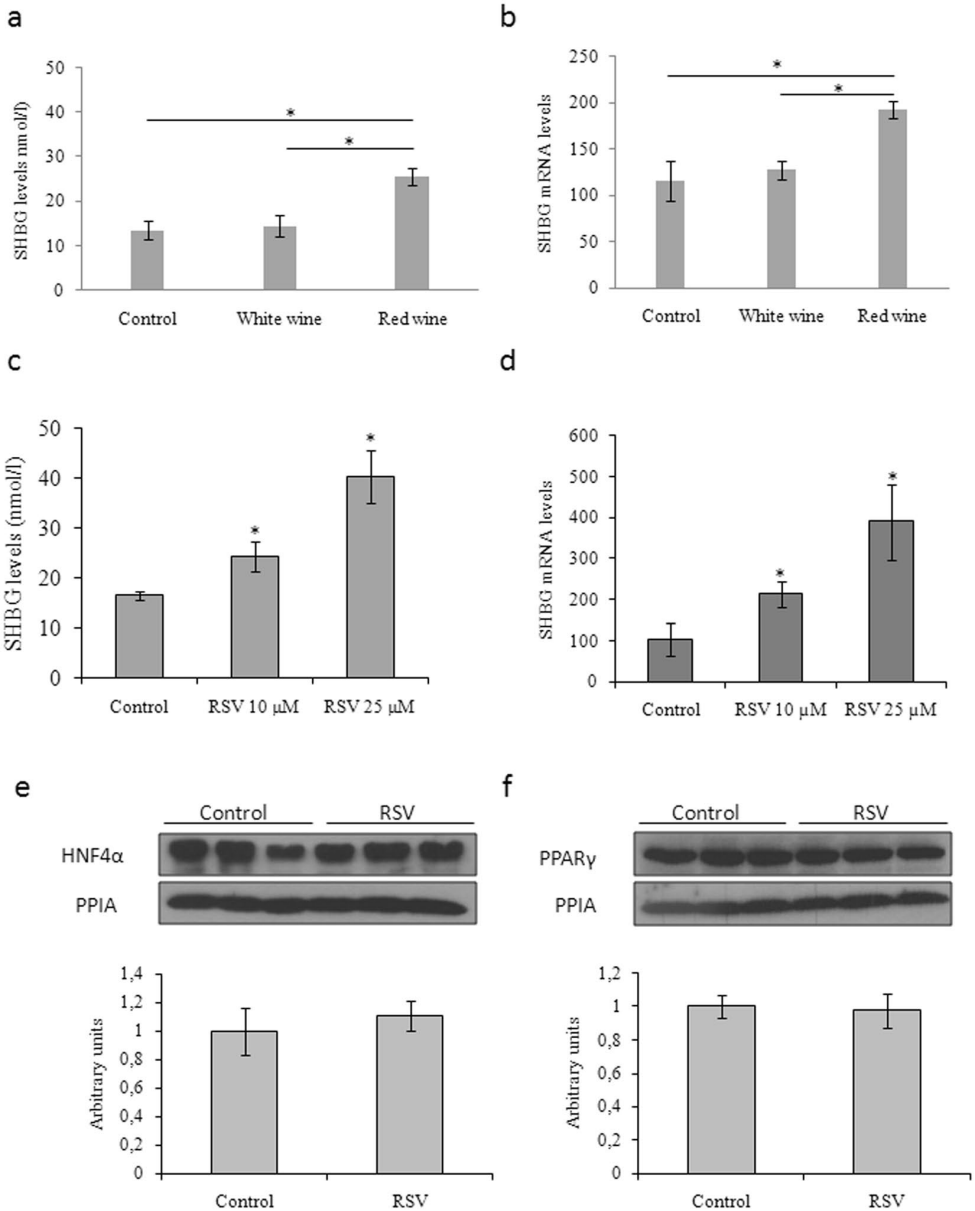
Resveratrol, a red wine polyphenol, increases SHBG production in HepG2 cells. (**a**) SHBG accumulation in the medium of HepG2 cells treated daily with vehicle, white wine (1 µl/ml) or red wine (1 µl/ml) was measured using an ELISA. (**b**) Analysis of SHBG mRNA levels in HepG2 cells treated as in A. Human 18 S (h18S) rRNA was amplified as a control. (**c**) SHBG accumulation in the medium of HepG2 cells treated daily with vehicle or resveratrol 10 µM or 25 µM was measured using an ELISA. (**d**) Analysis of SHBG mRNA levels in HepG2 cells treated as in C. Human 18 S (h18S) rRNA was amplified as a control. Data points are shown as mean ± SEM of triplicates. *p < *0.05* when compared with the control. (**e**) HNF-4α protein levels were measured by Western blotting using PPIA as a housekeeping reference protein in control and resveratrol (25 µM) treated HepG2 cells. Data points are mean ± SEM. (**f**) f PPARγ protein levels were measured by Western blotting using PPIA as a housekeeping reference protein in HepG2 cells treated as in (**e**). Data points are mean ± SEM.

The effects of daily supplementation of diluted (1/1,000) red wine or white wine on the SHBG production by HepG2 cells in culture were examined over 5 days. The trans-resveratrol content of the red and white wine used in this experiment was 5.9 mg/ml and 0.1 mg/ml, respectively. We measured the resveratrol concentration of the wines used in our experiments using an HPLC. As a standard we used pure trans-resveratrol. The resveratrol content of the wines was measured at 306 nm and 320 nm, since the cis-resveratrol has less absorption at 320 nm than 306 nm and the ratio 306/320 of the red wine was 1, we assumed that all resveratrol in the red wine was trans-resveratrol (Figure S1a,b, Supplementary Information)^51^. The results indicate that red wine treatment increased SHBG production when compared with white wine treated or control cells (Fig. 1a). Moreover, red wine treatment also increased SHBG mRNA levels significantly when compared with white wine treated or control HepG2 cells (Fig. 1b).

To test if resveratrol contributed to the ability of red wine to increase SHBG production by HepG2 cells, we treated the cells for 3 days with two concentrations of resveratrol (10 µM and 25 µM) in the media. This showed that SHBG and its mRNA levels were increased in a dose dependent manner after 3 days treatment with resveratrol when compared to control HepG2 cells (Fig. 1c,d).

To explore the molecular mechanisms by which resveratrol increased SHBG production in our HepG2 cell experiments, we examined the cell content of HNF-4α and PPARγ in control and resveratrol (25 µM) HepG2 cells, because they are key transcription factors involved in controlling *SHBG* expression in liver cells^8,9^. This showed that resveratrol treatment did not change HNF-4α or PPARγ levels under the experimental conditions (Fig. 1e,f).

It has been previously shown that resveratrol treatments at µM concentrations could induce cytotoxicity in cell cultures^52,53^. Therefore cell morphology and cell viability have been examined after resveratrol treatment to rule out any cytotoxic effect in our experiments. The results showed that resveratrol treatment did not induce any cytotoxic effects altering cell growth or morphological changes in HepG2 cells (Fig. S2a,b, Supplementary Information).

### Resveratrol does not increase SHBG production in human SHBG transgenic mice

**Figure 2:**
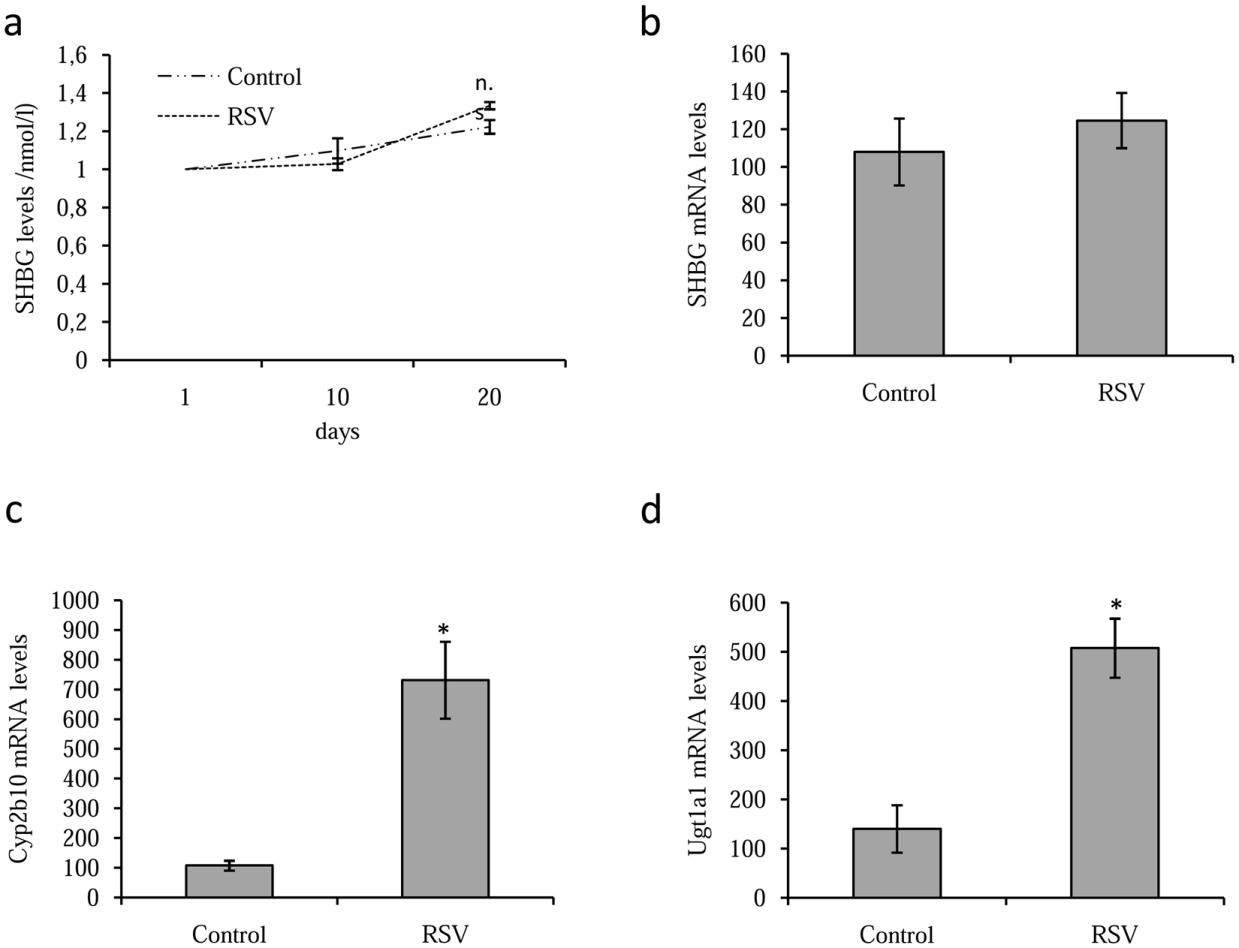
Resveratrol did not increase SHBG production in human SHBG transgenic mice. (**a**) SHBG plasma levels in human *SHBG* transgenic mice treated daily with vehicle or resveratrol (60 µg/ml) orally for 20 days. Data points are mean ± SEM of triplicate measurements. (**b**) Analysis of hepatic SHBG mRNA levels human *SHBG* transgenic mice treated as in (**a**). (**c**) Analysis of hepatic Cyp2b6 mRNA in human *SHBG* transgenic mice treated as in a. Human 18S (h18S) rRNA was amplified as a control. (**d**) Analysis of hepatic Ugt1a1 in human *SHBG* transgenic mice treated as in A. Human 18S (h18S) rRNA was amplified as a control. Data points are shown as mean ± SEM of triplicates. *p < *0.05* when compared with the control.

We next examined whether resveratrol treatment increases hepatic SHBG production *in vivo*. To do so, 4 male human *SHBG* expressing transgenic mice were given resveratrol (60 µg/ml) or vehicle in the drinking water for 20 days. Unexpectedly, plasma SHBG levels did not increase significantly by day 10 or day 20 after treatment (Fig. 2a), nor did hepatic SHBG mRNA levels (Fig. 2B), when compared with the vehicle treated control mice.

To verify that the resveratrol-treatment had been effective, we also analyzed hepatic Cyp2b10 and Ugt1a1 mRNA levels, because they are known to respond to resveratrol treatment in mice^28,54,55^. This showed that human *SHBG* transgenic mice treated with resveratrol had higher Cyp2b10 and Ugt1a1 mRNA levels when compared control mice (Fig. 2c,d).

### Human CAR mediates resveratrol-induced increases in SHBG production by HepG2 cells

Since resveratrol treatment of HepG2 cells increased SHBG production without altering the cellular levels of HNF-4α or PPARγ, we considered other transcription factors that might mediate the effects of resveratrol on *SHBG* gene expression. A potential candidate was CAR as it is known to bind resveratrol^34^ and because it has the potential to bind DR1 motifs in the human *SHBG* promoter that interact with transcription factors in liver nuclear extracts^46^, including HNF-4α or PPARγ^8,9^. Because the affinities of human and mouse CAR for ligands are known to differ^48–50^, HepG2 cells were first stably transfected with an empty vector or with human or mouse CAR expression vectors. When human CAR (hCAR) and mouse CAR (mCAR) mRNA levels were measured in the cells transfected with hCAR or mCAR expression vectors both increased when compared with the empty vector transfected HepG2 cells (Figure S3a, Supplementary Information). The overexpression of hCAR was further corroborated by western blot of CAR in empty vector and hCAR stably transfected HepG2 cells (Figure S3b, Supplementary Information).

**Figure 3:**
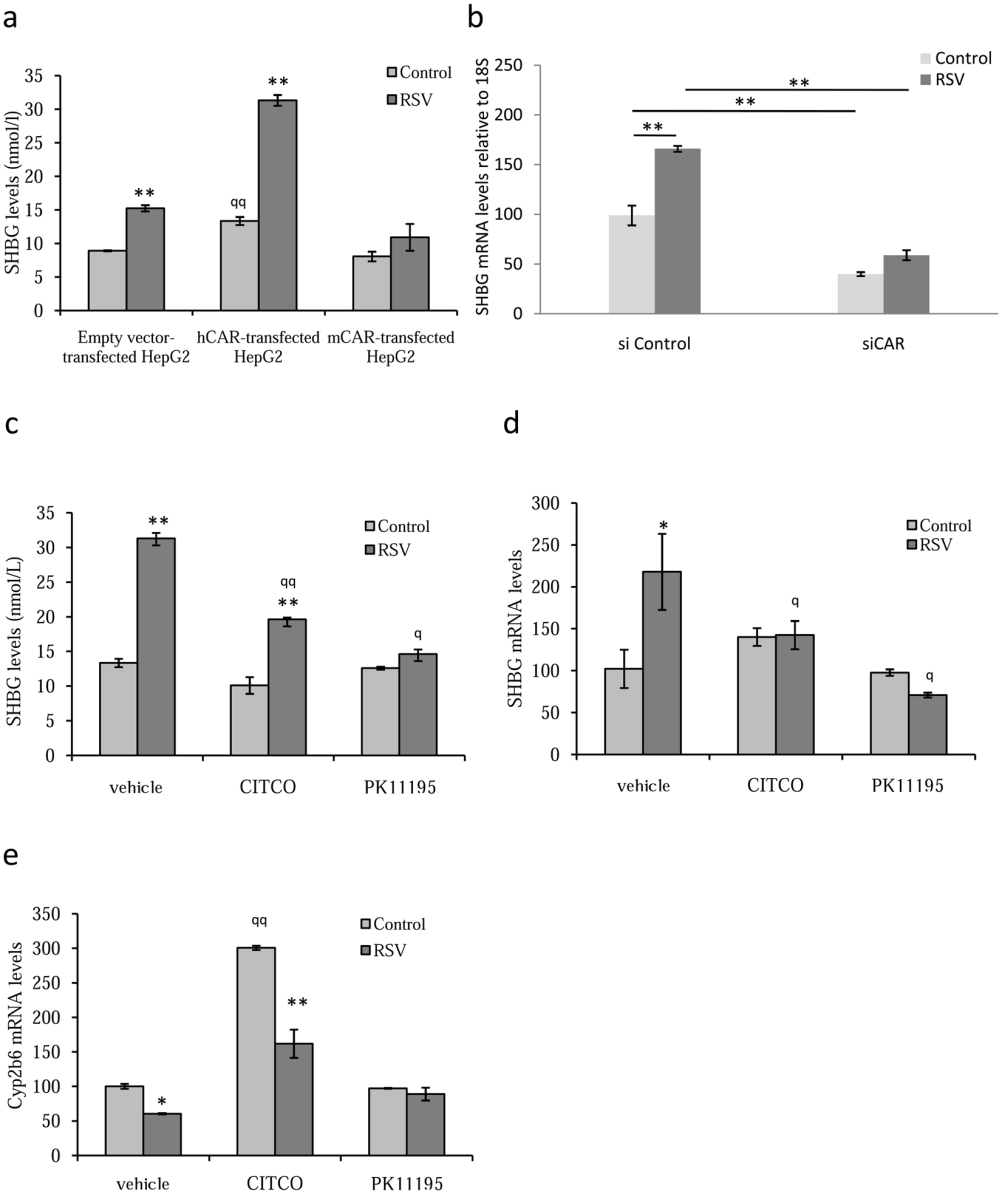
Human CAR is involved in resveratrol-induced increase in SHBG production in HepG2 cells. (**a**) SHBG accumulation in the medium of HepG2, hCAR or mCAR-transfected cells treated daily with vehicle or resveratrol (25 µM) for 3 days. Data points are shown as mean ± SEM of triplicates. **p < *0.01* when compared control *vs* resveratrol. ^*qq*^*p* < *0.01* when compared EV *vs* hCAR cells treated with vehicle (**b**) Analysis of SHBG mRNA expression in siRNA control or siRNA CAR transfected HepG2 cells in the presence or absence of resveratrol. Human 18 S (h18S) rRNA was amplified as a control. Data points are shown as mean ± SEM of triplicates. **p < *0.01*. (**c**) SHBG accumulation in the medium of hCAR-transfected cells treated daily with vehicle or resveratrol (25 µM) in the presence or absence of CITCO (1 µM) or PK11195 (10 µM) for 3 days. Data points are shown as mean ± SEM of triplicates. **p < *0.01* when compared vehicle *vs* resveratrol. ^*q*^*p* < *0.05* and ^*qq*^*p* < *0.01* when compared vehicle *vs* CITCO or PK11195. (**d**) Analysis of SHBG mRNA expression in hCAR-transfected cells treated as in B. Human 18 S (h18S) rRNA was amplified as a control. Data points are shown as mean ± SEM of triplicates. **p < *0.01* when compared vehicle *vs* resveratrol. ^*q*^*p* < *0.05* and ^*qq*^*p* < *0.01* when compared vehicle *vs* CITCO or PK11195. (**e**) Analysis of Cyp2b6 mRNA expression in hCAR-transfected cells treated as in B. Human 18 S (h18S) rRNA was amplified as a control. Data points are shown as mean ± SEM of triplicates. **p < *0.01* when compared vehicle *vs* resveratrol. ^*q*^*p* < *0.05* and ^*qq*^*p* < *0.01* when compared vehicle *vs* CITCO.

Next we treated the hCAR, mCAR or empty expression vector-transfected HepG2 cells with 25 µM resveratrol in the culture medium for 3 days. This showed that resveratrol treatment increased SHBG production in the empty vector and hCAR transfected HepG2 cells, however there was a much greater increase in SHBG production in resveratrol vs control hCAR transfected cells when compared with empty vector-transfected cells (Fig. 3a). Remarkably we found no increase in SHBG production after resveratrol treatment in mCAR-transfected HepG2 cells when compared to control cells (Fig. 3a). To further support these findings siRNA experiments to knockdown CAR expression in HepG2 cells were performed in order to determine whether the CAR downregulation could abolish the resveratrol induced SHBG production. We first showed that CAR siRNA treatment was effective in reducing both CAR mRNA and protein levels when compared with siRNA control treated HepG2 cells (Figure S3c, Supplementary Information). We next repeated the CAR knockdown experiments in the presence or absence of resveratrol (10 µM). The results showed that resveratrol induced increase expression of SHBG in siRNA control treated cells but this effect was abrogated when the cells were treated with CAR siRNA (Fig. 3B).

To ascertain that resveratrol acts via hCAR to increase SHBG production in hCAR transfected HepG2 cells, we treated hCAR transfected cells with resveratrol in the presence or absence of 1 µM CITCO, as a hCAR agonist, or 10 µM PK11195, as a hCAR antagonist, in the culture medium. The data showed that resveratrol increased SHBG production when compared with control vehicle-treated cells, while treatment with CITCO appears to dampen the RSV-induced increase in SHBG production and PK11195 treatment completely blocked it (Fig. 3c). These results were confirmed when SHBG mRNA levels were analyzed at the end of the treatment period (Fig. 3d). We also analyzed *Cyp2b6* expression levels because it is known to increase after CITCO treatment^48^. As expected, we found a very significant increase in Cyp2b6 mRNA levels in CITCO treated control cells while this was abrogated in cells co-treated with resveratrol (Fig. 3e). Moreover PK11195 treatment reduced the CITCO-induced increase in Cyp2b6 mRNA levels (Fig. 3e).

**Figure 4:**
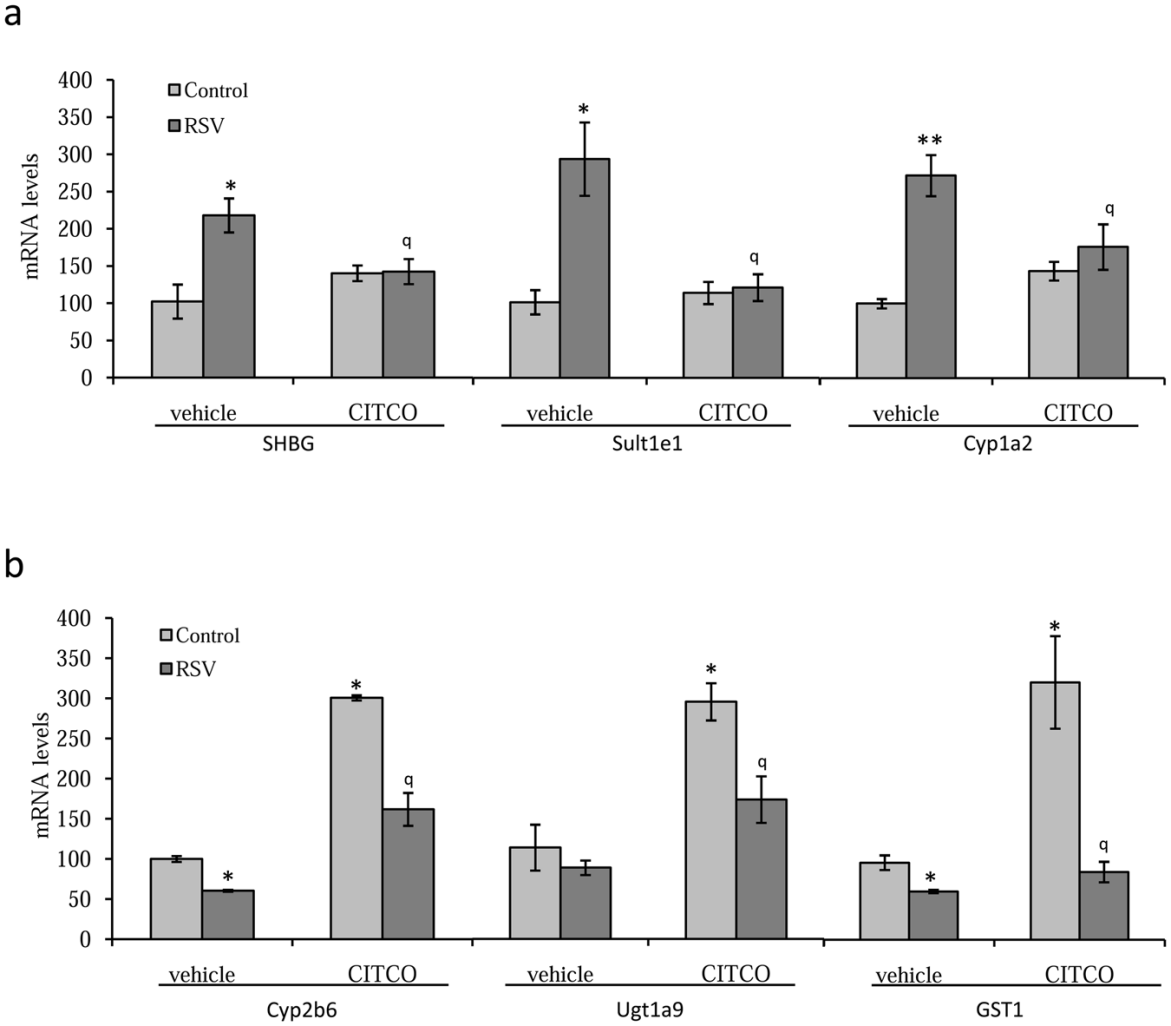
Resveratrol and CITCO regulate different set of genes through hCAR in HepG2 cells. (**a**) Analysis of SHBG, Sult1e1 and Cyp1a2 mRNA expression in hCAR-transfected HepG2 cells treated with vehicle or resveratrol (25 µM) in the presence or absence of CITCO (1 µM) for 3 days. Human 18S (h18S) rRNA was amplified as a control. (**b**) Analysis of Cyp2b6, Ugt1a9 and GST1 mRNA expression in hCAR-transfected HepG2 cells treated as in E. Human 18S (h18S) rRNA was amplified as a control. Data points are shown as mean ± SEM of triplicates. **p < *0.01* when compared vehicle *vs* resveratrol. ^*q*^*p* < *0.05* and ^*qq*^*p* < *0.01* when compared vehicle *vs* CITCO.

Our results suggested that resveratrol and CITCO act as CAR agonists regulating a different set of genes. To test this hypothesis, we repeated the resveratrol treatments in the presence or absence of CITCO and analyzed the expression of SHBG, Cyp2b6, Sult1e1, Ugt1a9, Cyp1a2 and GST1 in hCAR-transfected cells. The results showed that *SHBG*, *Sult1e1*, and *Cyp1a2* genes respond to resveratrol treatment and not to CITCO, when compared with control cells. The CITCO co-treatment blocked the increase in mRNA levels of these genes induced by resveratrol (Fig. 4a). In contrast, *Cyp2b6*, *Ugt1a9* and *GST1* genes did not respond to resveratrol treatment and showed an increased expression after CITCO treatment. However, resveratrol co-treatment reduced the increase in mRNA levels of these genes induced by CITCO treatment (Fig. 4b).

### Human CAR binds to a DR-1 element in the human SHBG proximal promoter in HepG2 cells

**Figure 5:**
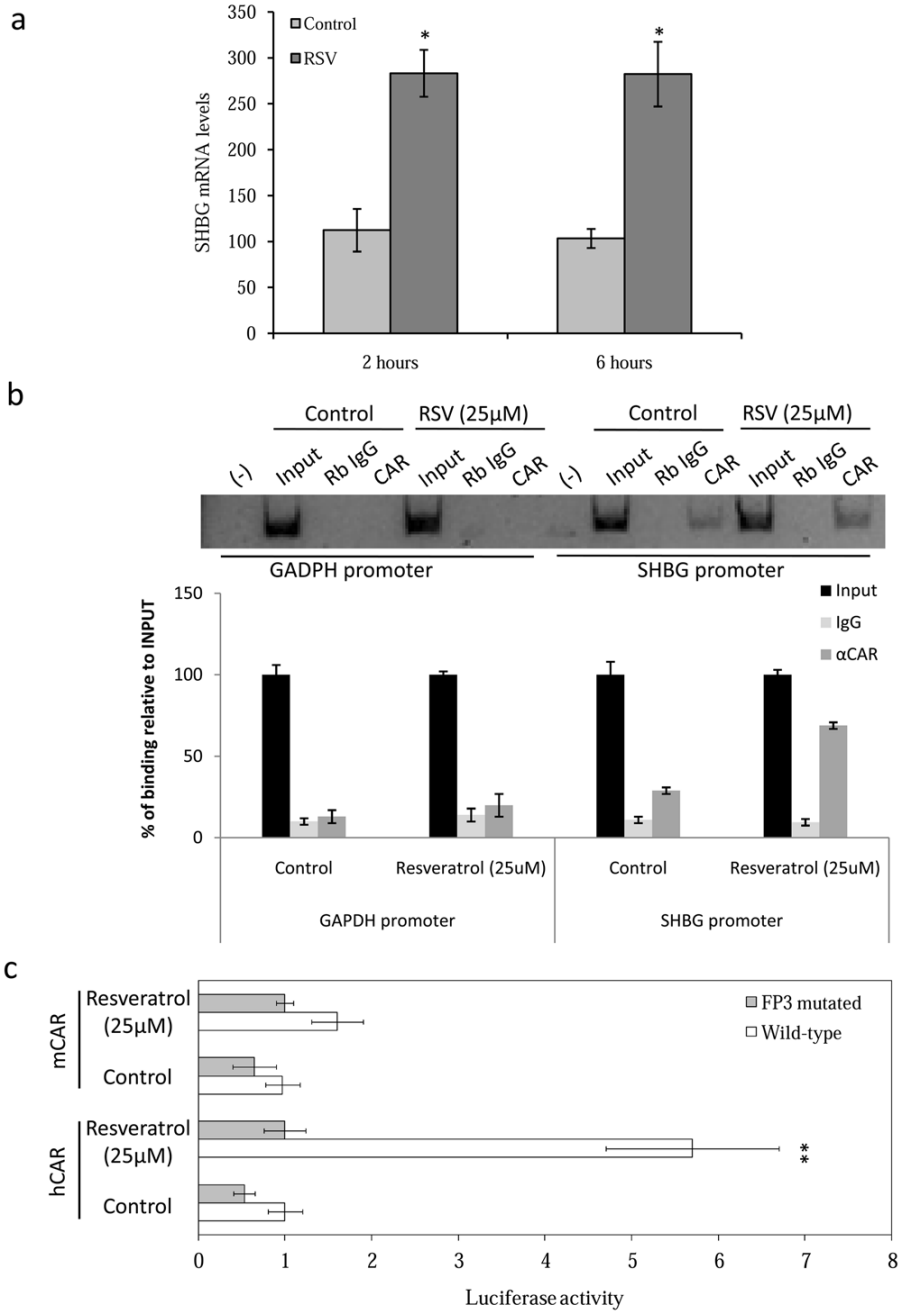
Resveratrol increases SHBG production through hCAR binding to the DR-1 motif present in the human SHBG proximal promoter. (**a**) Analysis of SHBG mRNA expression in hCAR-transfected HepG2 cells treated with vehicle or resveratrol (25 µM) during 2 and 6 hours. Human 18 S (h18S) rRNA was amplified as a control. Data points are shown as mean ± SEM of triplicates. *p < *0.05* when compared with the control. (**b**) ChIP assay of CAR binding to the human *GAPDH* and *SHBG* promoters in vehicle and resveratrol treated hCAR-transfected cells. Nonspecific rabbit IgGs were used in ChIP reactions to control for nonspecific immunoprecipitation. Positive PCR controls of sheared genomic DNA templates indicated the integrity of the input DNA used in the ChIP reactions. ChIP results were quantified by real-time PCR. (**c**) The activities of a wild-type or mutated (to disrupt the consensus DR-1 sequence at 70 bp from the start site of transcription) human *SHBG* promoter-driven luciferase reporter plasmids were analyzed in HepG2 cells in the presence of hCAR or mCAR expression vectors after treatment with vehicle (control) or resveratrol (25 µM). Data points are mean ± SEM of triplicate measurements. **P < 0.01 compared with the control.

To determine if resveratrol increases SHBG mRNA levels directly, we repeated the standard treatments of HepG2 cells with or without resveratrol and analyzed the SHBG mRNA levels 2 and 6 hours later. The results show that resveratrol increased SHBG mRNA levels significantly at both time points when compared with control cells (Fig. 5a).

To determine if resveratrol stimulates hCAR binding to the consensus DR-1 element (GGGTCAAGGGTCA) located about 70 bp upstream of the transcriptional start site of the *SHBG* proximal promoter, we performed chromatin immunoprecipitation assays (ChIP) using DNA/protein complexes from HepG2 cells treated with or without resveratrol (25 µM). This indicated that the *SHBG* promoter had an increasing bounding of hCAR in resveratrol-treated cells (Fig. 5B). As negative control, we used the GAPDH promoter, which was not amplified in either resveratrol-treated or untreated cells; these results were quantified by real-time PCR (Fig. 5b).

Because CAR interacts with DR-1 elements in different promoters^46^, and the first 299 bp of the human *SHBG* promoter contains a perfect consensus DR-1 sequence at 70 bp from the start site of transcription^8^, we performed luciferase reporter gene assays in HepG2 cells using wild-type or mutated DR-1 *SHBG* proximal promoter constructs co-transfected with either hCAR or mCAR, and treating them as previously with or without resveratrol. This showed that resveratrol increased human *SHBG* promoter activity in hCAR co-transfected cells when compared with the control cells (Fig. 5c), but did not change human *SHBG* promoter activity in the presence of mCAR (Fig. 5c). Importantly, resveratrol did not increase the activity of a human *SHBG* promoter with a mutated DR-1 element in the presence of hCAR or mCAR (Fig. 5c).

### Resveratrol increases SHBG levels in human SHBG/humanized CAR double transgenic mice

**Figure 6:**
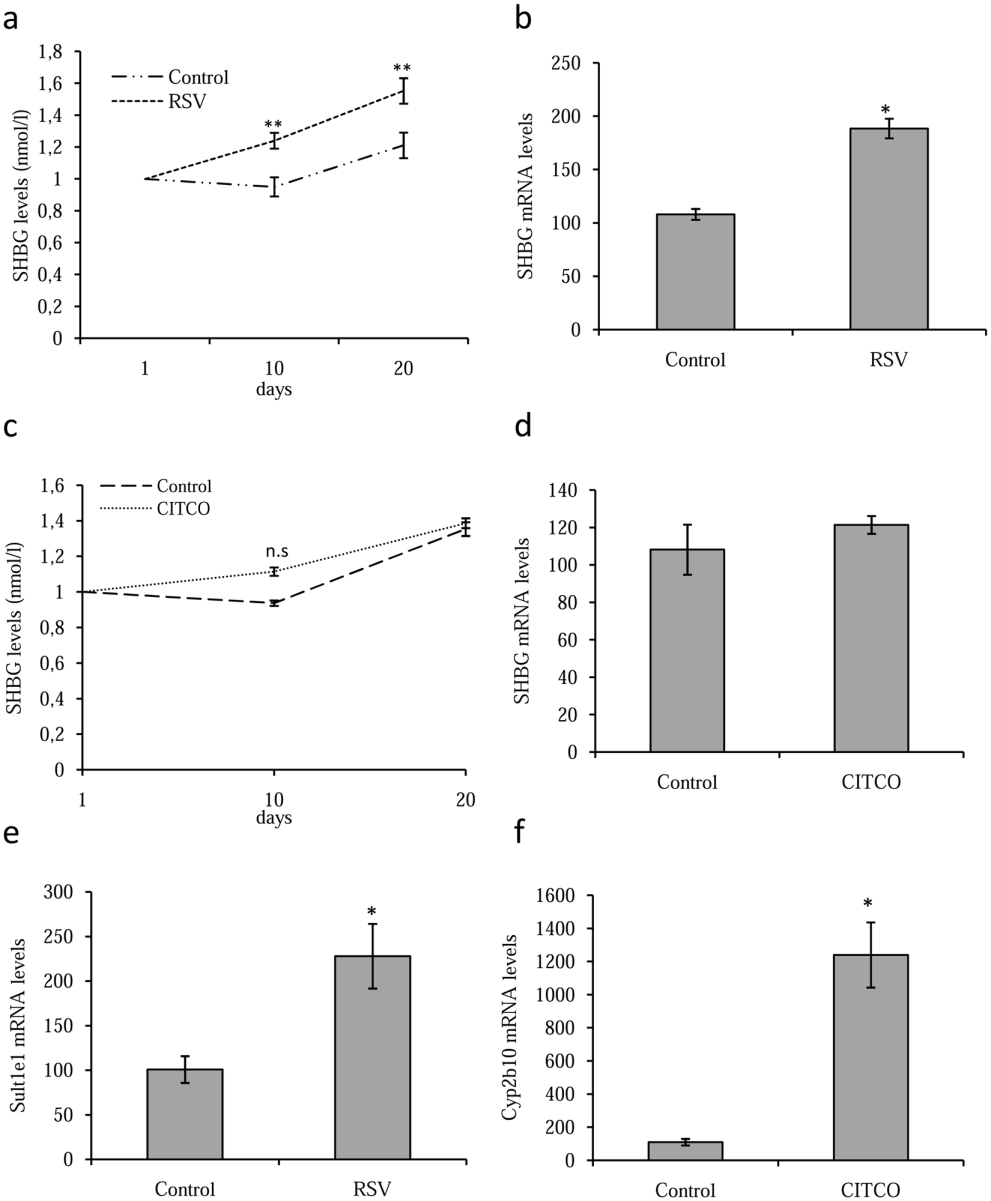
Resveratrol increases SHBG production in human SHBG/humanized CAR double transgenic mice. (**a**) SHBG plasma levels in human *SHBG*/humanized *CAR* mice treated daily with vehicle or resveratrol (60 µg/ml) orally for 20 days. Data points are mean ± SEM of triplicate measurements. (**b**) Analysis of hepatic SHBG mRNA levels human *SHBG*/humanized *CAR* mice treated as in A. Mouse 18 S (m18S) rRNA was amplified as a control. (**c**) SHBG plasma levels in human *SHBG*/humanized *CAR* mice treated daily with vehicle or CITCO (60 µg/ml) orally for 20 days. Data points are mean ± SEM of triplicate measurements. (**d**) Analysis of hepatic SHBG mRNA levels human *SHBG*/humanized *CAR* mice treated as in c. Mouse 18 S (m18S) rRNA was amplified as a control. (**e**) Analysis of hepatic Sult1e1 mRNA in human *SHBG*/humanized *CAR* mice treated as in a. Human 18 S (h18S) rRNA was amplified as a control. Mouse 18 S (m18S) rRNA was amplified as a control. (**f**) Analysis of hepatic Cyp2b10 in human *SHBG*/humanized *CAR* mice treated as in A. Mouse 18 S (m18S) rRNA was amplified as a control. Data points are shown as mean ± SEM of triplicates. *p < *0.05* when compared with the control.

Based on our findings in HepG2 cells that hCAR mediates an increase in human SHBG production in response to resveratrol treatment, while mCAR does not, we generated a double transgenic mouse line by crossing humanized *SHBG* and *CAR* transgenic mice, and treated these animals *ad libitum* for 20 days with resveratrol (60 µg/ml), CITCO (60 µg/ml) or vehicle (ethanol at 0.001%) in the drinking water. This showed that resveratrol treatment increased plasma SHBG levels by days 10 and 20 when compared with vehicle treated control mice (Fig. 6a). Moreover, resveratrol treatment increased SHBG mRNA levels when compared with control mice (Fig. 6b). By contrast, CITCO treatment did not significantly increase plasma SHBG levels in the transgenic mice when compared to control mice (Fig. 6c) and did not affect the SHBG mRNA levels (Fig. 6d). We also found that resveratrol treatment increased Sult1e1 mRNA levels when compared with the control mice (Fig. 6e) while CITCO treatment increased Cyp2b10 mRNA levels when compared with the controls (Fig. 6f).

### SHBG mRNA levels correlated with those of CAR in human liver biopsies

**Figure 7:**
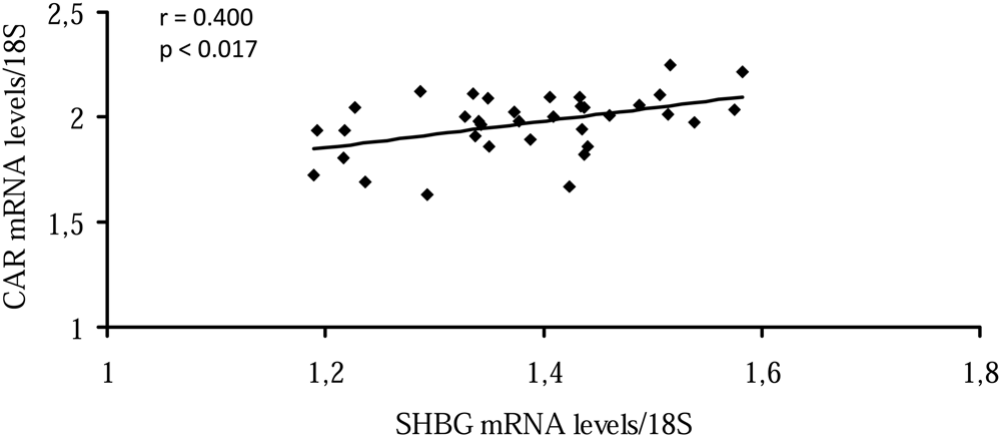
SHBG correlation with CAR mRNA levels in human liver biopsies. Correlation between SHBG mRNA levels and CAR mRNA levels in human liver biopsies (n = 35).

To examine whether the expression of *SHBG* and *CAR* are related to each other in human liver, we analyzed SHBG and CAR mRNA levels in human liver biopsies (n = 35), and found that SHBG mRNA levels are positively and significantly correlated with CAR mRNA levels (Fig. 7).

## Discussion

Epidemiological studies have shown that red wine, an important constituent of the Mediterranean diet, has beneficial effects on metabolic syndrome and cardiovascular disease^16–20^. It is also well established that low plasma SHBG levels are present in subjects suffering the metabolic syndrome and predict a higher risk of suffering cardiovascular disease^2–7^. In a pilot study we observed that red wine increased SHBG production in HepG2 cells while white wine did not. Moreover, we found that resveratrol, a polyphenol that is more abundantly in red wine than in white wine^24,26,32^ increased SHBG production by HepG2 cells. However, it is important to mention that diluted red wine with an amount of resveratrol at the nM range it was more effective in increasing SHBG production in HepG2 cells than pure resveratrol at µM range. A possible explanation for these differences is that the red wine contains other substances with capacity to upregulate the SHBG production. Another consideration that we have to take into account is that other antioxidants compounds of red wine could give more stability to the resveratrol than the pure resveratrol^56,57^. This could be the reason why resveratrol at µM concentration is currently used in the literature^58–61^. There are few reports of the effects of red *vs* white wine on plasma SHBG levels: a 5% reduction in plasma SHBG levels was observed in premenopausal drinking red wine *vs* white wine, but the red wine used in that study contained only 0.9 mg/L of resveratrol^62^. By contrast, plasma SHBG was recently reported to increase by about 10% after resveratrol treatment in postmenopausal women^63^. In light of the latter report and our observation that resveratrol increased *SHBG* expression in human HepG2 cells, the fact that resveratrol failed to increased plasma SHBG levels in human *SHBG* transgenic mice was suprising, especially as Cyp2b10 and Ugt1a1 mRNA increased in their livers, and this suggested a species difference in hepatic transcription factors responsible for resveratrol mediated effects on *SHBG* gene expression.

To study the mechanisms involved in *SHBG* regulation by resveratrol, we initially analyzed two key transcription factors known to control hepatic *SHBG* expression, namely HNF-4α and PPARγ^8,9^, but their levels did not change in resveratrol-treated HepG2 cells. We therefore focused our attention to CAR because it is know to bind resveratrol^34^; could potentially interact with a consensus DR-1 element in the human *SHBG* promoter^49^ and exhibits differences in its activities between human and mice^47–50^. Our experiments in human HepG2 cells showed that resveratrol increased SHBG production through human CAR but not mouse CAR and provided and explanation for why resveratrol did not increase plasma SHBG levels in our *in vivo* experiments using human *SHBG* transgenic mice, despite the fact that it did increase Cyp2b10 and Ugt1a1 mRNA in their livers as reported by others^28,54,55^.

**Figure 8:**
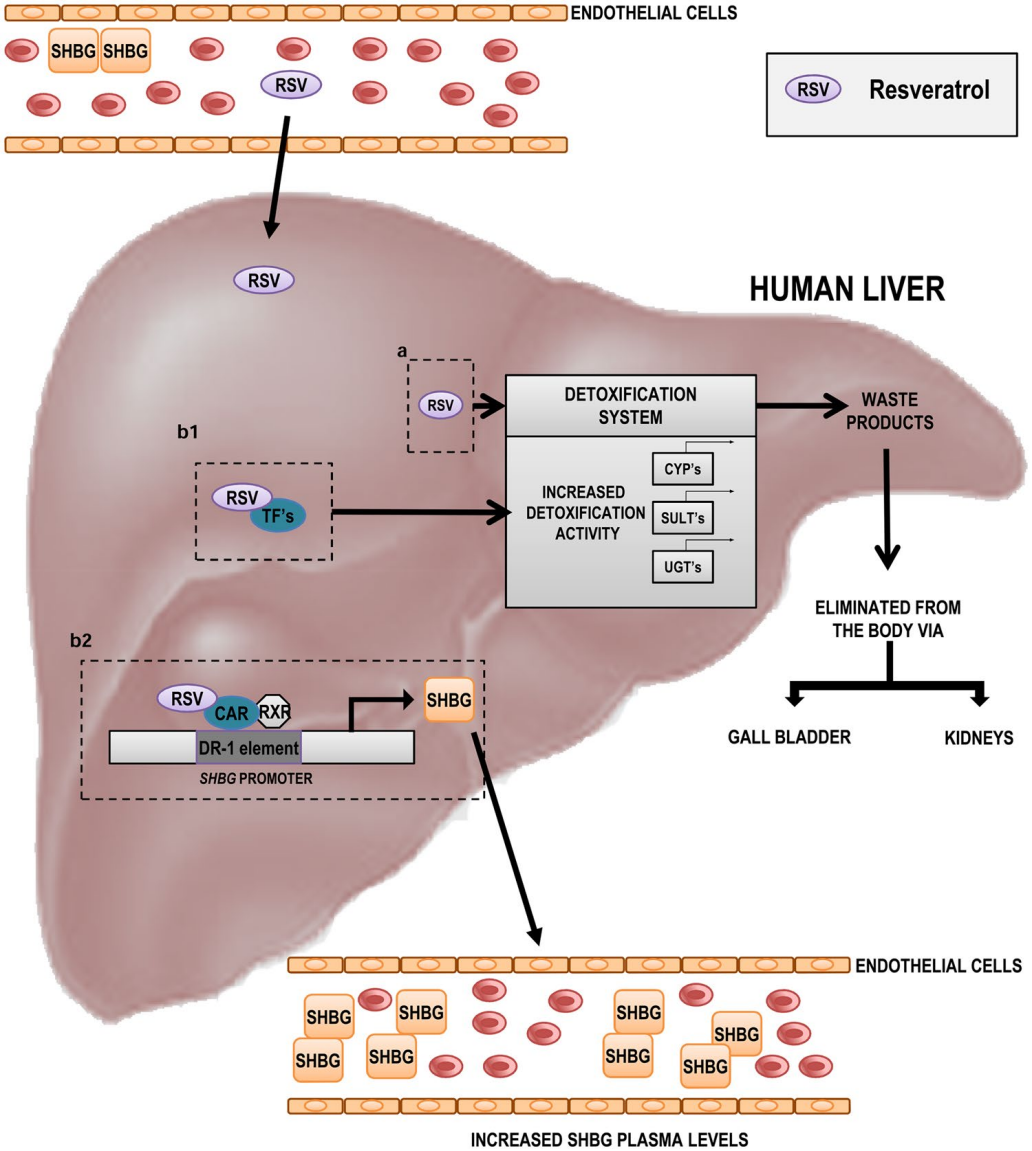
Resveratrol increases hepatic SHBG production through constitutive androstane receptor. Circulating resveratrol enters the liver and (**a**) it suffers degradation by the activity of different enzymes, such as CYP’s, SULT’s and UGT’s, (**b1**) it binds to different transcription factors (TFs) regulating the expression of drug/detoxification genes, (**b2**) through the binding to CAR it increases SHBG production.

We have found that resveratrol increases SHBG mRNA levels within two hours of treatment, and that this effect is mediated by CAR interacting with a consensus DR-1 element in the human *SHBG* proximal promoter (Fig. 8). There is precedence for this because CAR competes with HNF-4α for binding to DR-1 motifs in the *CYP7A1*, *CYP8B1*, and *PEPCK* promoters^46^. The region of the human *SHBG* proximal promoter that contains this consensus DR-1 element is remarkable in that it appears to be responsible for controlling its tissue specific expression^8^ as well as regulating responses to numerous nutritional^15,64^ and hormonal^65,66^ stimuli. The DR-1 element through which CAR appears to mediate the effects of resveratrol on *SHBG* expression also binds COUP-TF, HNF-4α^8^ and PPARγ^9^. It may also bind other members of the PPAR family, as well as RXR homodimers or heterodimers with other NHRs, including RAR, LXR, PPARα, FXR, and PXR many of which are metabolic, drug or xenobiotic sensors^67^. Thus, unlike the DR-1 element positioned close to the *SHBG* transcription start site, and which functions through interactions with COUP-TF and HNF-4α as the main transcriptional “on-off” switch in the liver^8^, the upstream DR-1 element that interacts with CAR may represent a “fine-tuner” that responds to a number of related NHRs many of which regulate responses to metabolic disturbances during disease or after exposure to drugs or environmental xenobiotic ligands. We were therefore not surprised by the positive correlations observed between both SHBG and CAR mRNA levels in human liver biopsies.

The increases in plasma SHBG levels we observed in human *SHBG* and *CAR* double transgenic mice after resveratrol supported the conclusions from our experiments in HepG2 cells, and may explain the increases in plasma SHBG levels observed in postmenopausal women after oral resveratrol treatment^63^. However, our study has several limitations; first our results showed that red wine is more effective in increasing SHBG than pure resveratrol, needing one order of magnitud higher (µM) of resveratrol than red wine (nM) to induce similar effects on SHBG production. A potential explanation of this finding is that other substances apart from resveratrol contained in the red wine could increase the SHBG production. Second, the study of *SHBG* transcriptional regulation by using the human *SHBG* transgenic mice is not ideal even though these mice contain in their *SHBG* transgenes 0.9 kb of the human *SHBG* promoter sequence.

The stimulatory effect of resveratrol on hepatic *SHBG* expression is particularly interesting because of the beneficial effects that red wine consumption has on reducing risk of the metabolic syndrome, type 2 diabetes and cardiovascular disease^22–28^, and because plasma SHBG levels are well known to be low in obese individuals at high risk for these diseases. While it is generally accepted that low plasma SHBG levels are a sensitive predictive biomarker of increased risk for cardiovascular disease^6,7^ and type 2 diabetes^3^ there is evidence that genetic differences associated with high versus low plasma SHBG levels protect or predispose individuals to type 2 diabetes, respectively^3^. If the latter is correct, it will be important to determine if the beneficial health effects of resveratrol might be mediated in part by increasing plasma SHBG levels *per se*. This is not unreasonable because changes in plasma SHBG levels have a pivotal role in controlling the balance between circulating androgens and estrogens, both of which are directly implicated as either risk factors or more directly in the etiology of the metabolic syndrome in men and women^68–71^. Thus, our discovery that resveratrol induced increases in plasma SHBG may represent a paradigm for therapeutic interventions that could reduce risk for many of the diseases associated with metabolic syndrome.

## Methods

### Subjects and samples

We recruited 35 (15 men and 20 women) subjects of Caucasian origin (body mass index between 32.61–53.31 kg/m^2^) with no evidence of metabolic disease other than obesity, and patients with type 2 diabetes were excluded. All patients had fasted overnight, at least 12 h before undergoing surgical procedure. Two experienced surgeons in abdominal surgery performed all the laparoscopically Roux-en Y gastric bypass procedures under standard anaesthesia at the University Hospital Vall d’Hebron (Barcelona, Spain). A liver biopsy was obtained from all obese patients included in the study and it was immediately frozen in liquid nitrogen and stored at −80 C. The Human Ethics Committee at the Hospital Vall d’Hebron approved the study protocol, all the following methods were performed in accordance with the relevant guidelines and regulations issued in this protocol. Informed written consent was obtained from all participants of this study.

### Animals

Human SHBG transgenic mice used in this study contain a 4.3-kb region of the human *shbg* locus, which includes approximately 0.9 kb of the promoter region together with the codifying region. These mice express the human SHBG in their livers which results in the presence of human SHBG in their bloods^72^. Humanized CAR mice (C57BL/6-*Nr1i3*^*tm1(NR1I3)Arte*^) were purchased from Taconic Biosciences (Hudson, US) and crossed with human *SHBG* transgenic mice for at least 5 generations. All mice used in this study were male. The human *SHBG*/Humanized *CAR* mice used in the experiments were homozygous for human CAR. Human *SHBG* transgenic mice were genotyped using an SHBG ELISA (Demeditec Diagnostics GmbH) and humanized *CAR* mice were genotyped by PCR using specific primers (5′-CTCAACTCCTCCCACATTCAG and 5′-TGCTCTTGACTAATGGGCTG) as previously described^73^.

Mice were maintained under standard conditions with food (Global Diet 2018, Harlan Interfauna Iberica, Barcelona, Spain) and water provided *ad libitum* and a 12 h light/dark cycle. Experimental procedures were approved by the Institutional Animal Use Subcommittees of Hospital Vall d’Hebron Research Institute and the Universitat Autonoma Barcelona. Animals were bred and all experimental methods were performed in accordance with the relevant guidelines and regulations issued in this protocol.

### Cell culture experiments

Cell culture reagents were from Life Technologies Inc (Invitrogen SA, Barcelona, Spain). HepG2 hepatoblastoma cells (catalog no. HB-8065; ATCC) were maintained in low glucose DMEM supplemented with 10% FBS and antibiotics. For experiments, HepG2 cells were cultured to 40–60% confluence prior to the addition of diluted red wine (1 µl/ml) (Vilafranca del Penedès, Spain), diluted white wine (1 µl/ml) (Rueda, Spain), resveratrol (25 or 50 µM) (Sigma-Aldrich), CITCO (1 µM) (Sigma-Aldrich) and PK11195 (10 µM) (Sigma-Aldrich). As vehicle controls in the experiments we used phosphate buffered saline (PBS), ethanol or DMSO (Sigma-Aldrich). All vehicle controls were added at the same amount in the media (0.001%) as the corresponding treatments. Resveratrol was dissolved in ethanol while CITCO and PK11195 were dissolved in DMSO.

Stable and transient transfections were performed using Lipofectamine 2000 following the manufacturer’s instructions (Invitrogen SA). HepG2 cells were stably transfected with empty vector (as a control), human or mouse CAR expression vectors. Cells were selected during 10 days by adding G418 (750 µg/ml) to the culture medium (Invitrogen SA). Transient transfections were performed using a wild-type or mutated (to disrupt the consensus DR-1 sequence at 70 bp from the start site of transcription) human *SHBG* promoter-driven luciferase reporter plasmids together with a pCMRenilla as control plasmid in the presence or absence of hCAR or mCAR expression vectors. Transient transfection experiments were performed for 48 h and the treatments with or without resveratrol (25 µM) were performed in the final 24 h of the experiment. Two days after transfection, luciferase and renilla activity were measured using the Dual-Luciferase Reporter Assay System (Promega, Barcelona, Spain). The siRNA experiments were carried out using HiPerfect Transfection Reagent (QIAGEN, Madrid, Spain) using a control siRNA (SR30005, OriGene, Rockville, USA) or CAR siRNA (SR306706, OriGene).

### Resveratrol Measurements

Resveratrol was measured in wine samples by HPLC-UV as previously described^51^ with some modifications. Briefly, wine samples were centrifuged at 20,000xg for 5 min at 4 °C and the 5 µl of supernatants were injected into an Acquity UPLC apparatus (Waters, Milford, MA, USA) using an Acquity UPLC BEH C18 column (100 × 2.1 mm, 130 Å pore, 1.7 µm particle, Waters). The components of the sample were resolved through an isocratic elution method using 75% of a saline buffer (20 mmol/l ammonium acetate, pH 5.6) and 25% acetonitrile at a flow rate of 0.4 ml/min. Elute optical absorbance was monitored at 306 nm and 320 nm. The quantification of resveratrol was based on the peak areas using external standards with known resveratrol concentrations.

### *In vivo* experiments

Human *SHBG* transgenic mice (n = 8) were divided randomly into two groups and given resveratrol (60 µg/ml) or the same amount of vehicle (ethanol, 0.001%) in the drinking water for 20 days. Saphenous vein samples were taken by for measurements of plasma SHBG levels immediately before the treatment and on days 10 and at the end of the treatment (day 20), when livers were taken for RNA and protein extraction.

In a second experiment, 28 humanized *SHBG/CAR* double transgenic mice were randomly divided into four groups, two groups of 10 that were given resveratrol (60 µg/ml) or vehicle (ethanol, 0.001%) in the drinking water during 20 days and two groups of 4 mice that were given CITCO (60 µg/ml) or vehicle (DMSO, 0.001%) in the drinking water during 20 days. Saphenous vein samples were taken for measurements of plasma SHBG levels immediately before the treatment and on days 10 and at the end of the treatment (day 20), when livers were taken for RNA and protein extraction.

### SHBG measurements

SHBG levels in culture medium from HepG2 cells and from mice plasma were measured using an ELISA (Demeditec Diagnostics GmbH).

### RNA analyses

Total RNA was extracted from HepG2 cells and mice livers using TRIzol reagent (Invitrogen SA). Reverse transcription (RT) was performed at 42 °C, for 50 min using 3 μg of total RNA and 200 U of Superscript II together with an oligo-dT primer and reagents provided by Invitrogen. An aliquot of the RT product was amplified in a 25-μl reaction using SYBRGreen (Invitrogen SA) with appropriate oligonucleotide primer pairs corresponding to human CAR, SHBG, HNF-4α, PPARγ, Cyp2b6, Cyp1a2, GST1, Sult1e1, Ugt1a1, Ugt1a9 mRNAs and mouse CAR, Cyp2b10, Ugt1a1, Sult1e1 mRNAs and an 18 S RNA as control (Table S1, Supplementary Information). Results were analyzed using the 7000 SDS program.

### Western Blot Analysis

HepG2 cell samples were homogenized in RIPA buffer with Complete™ protease inhibitor cocktail (Roche Diagnostics, Barcelona, Spain). Protein extracts were used for western blotting with antibodies against HNF-4α (C-19; catalog sc-6556; Santa Cruz Biotechnology Inc., Santa Cruz, CA, USA), PPARγ (H-100; catalog sc-7196; Santa Cruz Biotechnology Inc.), CAR (TA805288; Origene) and PPIA (SA-296; BIOMOL Int., Madrid, Spain). Specific antibody-antigen complexes were identified using the corresponding HRP-labeled goat anti-rabbit IgG and chemiluminescent substrates (Millipore) by exposure to x-ray film.

### Chromatin immunoprecipitation (ChIP) Assays

After treatment, HepG2 cells were used to perform ChIP assays with a ChIP-IT kit (Active Motif Inc.) as described previously^66^. The purified DNA was subjected to PCR amplification (35 cycles) using specific primers designed to amplify the human *GAPDH* and *SHBG* promoter (Table S1, Supplementary Information).

### Statistical analyses

Normal distribution of the variables was evaluated using the Kolmogorov-Smirnov test. Comparison of quantitative variables was performed by the Student’s *t* test or Mann–Whitney test according to the data distribution. All data are presented as means ± standard error of the mean. Significance was accepted at the level of *p* < 0.05. Pearson’s correlation coefficients were used to establish the association between SHBG mRNA levels and the CAR mRNA levels analyzed in human liver biopsies. For graphics a linear regression test was applied. Significance was accepted at the level of *p* < 0.05. Statistical analyses were performed with the SPSS statistical package (SPSS Inc, Chicago, Illinois).

## Electronic supplementary material

Supplementary Info
