# Author Correction: Resveratrol Increases Hepatic SHBG Expression through Human Constitutive Androstane Receptor: a new Contribution to the French Paradox

**DOI:** 10.1038/s41598-018-23159-y

**Published:** 2018-03-19

**Authors:** Cristina Saez-Lopez, Laura Brianso-Llort, J. Torres-Torronteras, Rafael Simó, Geoffrey L. Hammond, David M. Selva

**Affiliations:** 1 Diabetes and Metabolism Research Unit, Vall Hebron Institut de Recerca (VHIR). Universitat Autònoma de Barcelona and Biomedical Network Research Centre on Diabetes and Metabolic Diseases (CIBERDEM, ISCIII), Barcelona, Spain; 2 Research Group on Neuromuscular and Mitochondrial Diseases, Vall Hebron Institut de Recerca (VHIR). Universitat Autònoma de Barcelona and Biomedical Network Research Centre on Rare Diseases (CIBERER, ISCIII), Barcelona, Spain; 3 Cellular & Physiological Sciences, University of British Columbia, Vancouver, Canada

Correction to: *Scientific Reports* 10.1038/s41598-017-12509-x, published online 25 September 2017

The original version of this Article contained a typographical error in the spelling of the author J. Torres-Torronteras, which was incorrectly given as J. Torres-Torrenteras. This has now been corrected in the PDF and HTML versions of the Article and in the accompanying supplementary material.

