# Supplementary material for: Resveratrol Increases Hepatic SHBG Expression through Human Constitutive Androstane Receptor: a new Contribution to the French Paradox
Source: Sci Rep. 2017 Sep 25;7:12284. doi: 10.1038/s41598-017-12509-x (PMC5612985; doi:10.1038/s41598-017-12509-x)

# Resveratrol Increases Hepatic *SHBG* Expression through Human Constitutive Androstane Receptor: a new Contribution to the French Paradox

CRISTINA SAEZ-LOPEZ[1], LAURA BRIANSO-LLORT[1], JAVIER TORRES-TORRONTERAS[2], RAFAEL SIMÓ[1¶], GEOFFREY L. HAMMOND[3¶] AND DAVID M. SELVA[1¶]

[¶]**Address Correspondence to**: david.martinez.selva@vhir.org

**Table S1.** List of primers used for RT-PCR reactions and ChIP assay experiments.

| **Real-time PCR** | **Forward primer (5′-3′)** | **Reverse primer (5′-3′)** |
|---|---|---|
| hSHBG | GCTGATTATGGAGAGCAGAGG | GGTCATGACAGCGATAGGCT |
| hHNF-4α | GCTCCTCCTTCTGCTGCTGC | GGAAGAGCTTGAGACAGGCC |
| hPPARγ | TGAAGCTGAACCACCCTGAGT | GACCGTGTTCCGTGACAATC |
| hCAR | GAGCTGAGGAACTGTGTGGTA | CTTTTGCTGACTGTTCTCCTGAA |
| hCyp2b6 | GGAAAAAGAGAAATCCAACGC | CAGGAAGCCGTAGCGGAG |
| hSult1e1 | GGAAACAGCCACATCCTTTG | TTGCCACCTGAACTTCTTCC |
| hCyp1a2 | AGGGCTTGTTAATGGCAGTG | CTTCGTAAACCAGTGGCAGG |
| hGST1 | ATTGGCCTCCTGTATTCCTTGA | GTGCTCCGACAAATAGTCTGAAG |
| hUgt1a9 | ACCACAATTCCATGTTCTCCA | ACTATCCCAAACCCGTGATG |
| h18S | TAACGAACGAGACTCTGGCAT | CGGACATCTAAGGGCATCACAG |
| mCAR | CCTGCTGCCTAAGGGAAAC | TCTTCACTGGCCATGGTTTCTA |
| mCyp2b10 | TTGTTACTCTTAGCCAGGGGAC | GGAAGTTGCCACGGGACTTT |
| mUgt1a1 | AGATCTCTGGCAGCATCAGA | TCCAAGCATACTCAGCCAGT |
| mSult1e1 | GGAAACAGCCACATCCTTTG | TTGCCACCTGAACTTCTTCC |
| mCyp7a1 | CAGGGAGATGCTCTGTGTTCA | AGGCATACATCCCTTCCGTGA |
| mCyp2d9 | AGTCTCTGGCTTAATTCCTGAGGTT | CGCAAGAGTATCGGGAATGC |
| m18S | AGGGTTCGATTCCGGAGAGG | CAACTTTAATATACGCTATTGG |
| **ChIP** | **Forward primer (5′-3′)** | **Reverse primer (5′-3′)** |
| hSHBG promoter | CTAGACCTCAGGCCTGTGAATGC | GGCAGGCAGCCTTGCGTGTG |
| hGAPDH promoter | TACTAGCGGTTTTACGGGCG | TCGAACAGGACCAGCAGAGAGCGA |

Figure S1. (a) HPLC standard and calibration plot using trans-resveratrol measured at 306nm. (b) The resveratrol content of the wines was measured at 306 nm and 320 nm.

**a**

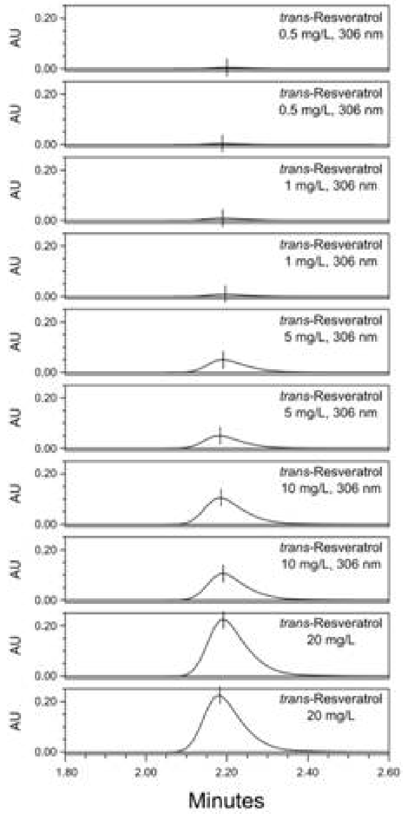


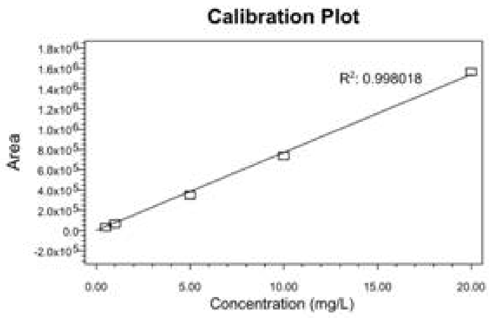


**Component Summary Table**
***trans*-Resveratrol**

| Sample name | RT (min) | Area | Concentration (mg/L) |
|---|---|---|---|
| Resveratrol 0.5 | 2.189 | 32879 | 0.5 |
| Resveratrol 0.5 | 2.200 | 32845 | 0.5 |
| Resveratrol 1 | 2.190 | 66945 | 1.0 |
| Resveratrol 1 | 2.196 | 67333 | 1.0 |
| Resveratrol 5 | 2.183 | 680231 | 5.0 |
| Resveratrol 5 | 2.191 | 348332 | 5.0 |
| Resveratrol 10 | 2.191 | 737143 | 10.0 |
| Resveratrol 10 | 2.185 | 736956 | 10.0 |
| Resveratrol 20 | 2.182 | 1566703 | 20.0 |
| Resveratrol 20 | 2.192 | 1566100 | 20.0 |

**b**

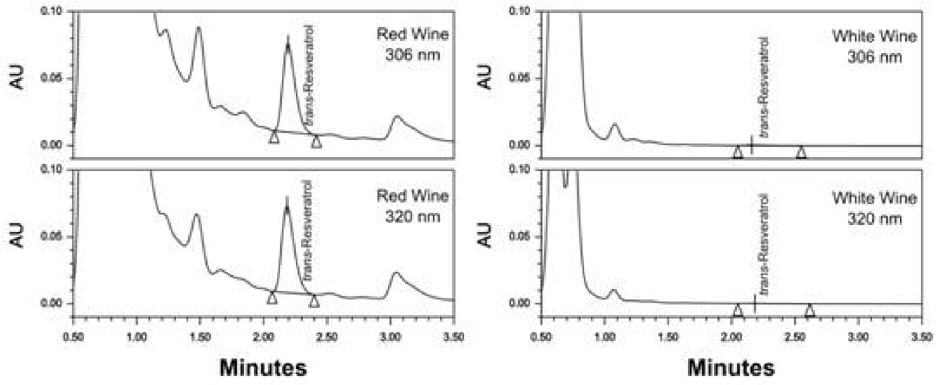


**Component Summary Table**
***trans*-Resveratrol**

| Sample name | Wavelenght (nm) | RT (min) | Area | Concentration (mg/L) | Ratio 306/320 |
|---|---|---|---|---|---|
| Red Wine | 306 | 2.194 | 453122 | 5.88 | 1.01 |
| Red Wine | 320 | 2.186 | 445130 | 5.83 | |
| White Wine | 306 | 2.160 | 5215 | 0.07 | 2.83 |
| White Wine | 320 | 2.185 | 1805 | 0.02 | |

Figure S2. (a) Cell morphology examination after 3 days resveratrol treatment in HepG2 cells. (b) Cell viability quantification after 3 days resveratrol treatment in HepG2 cells.

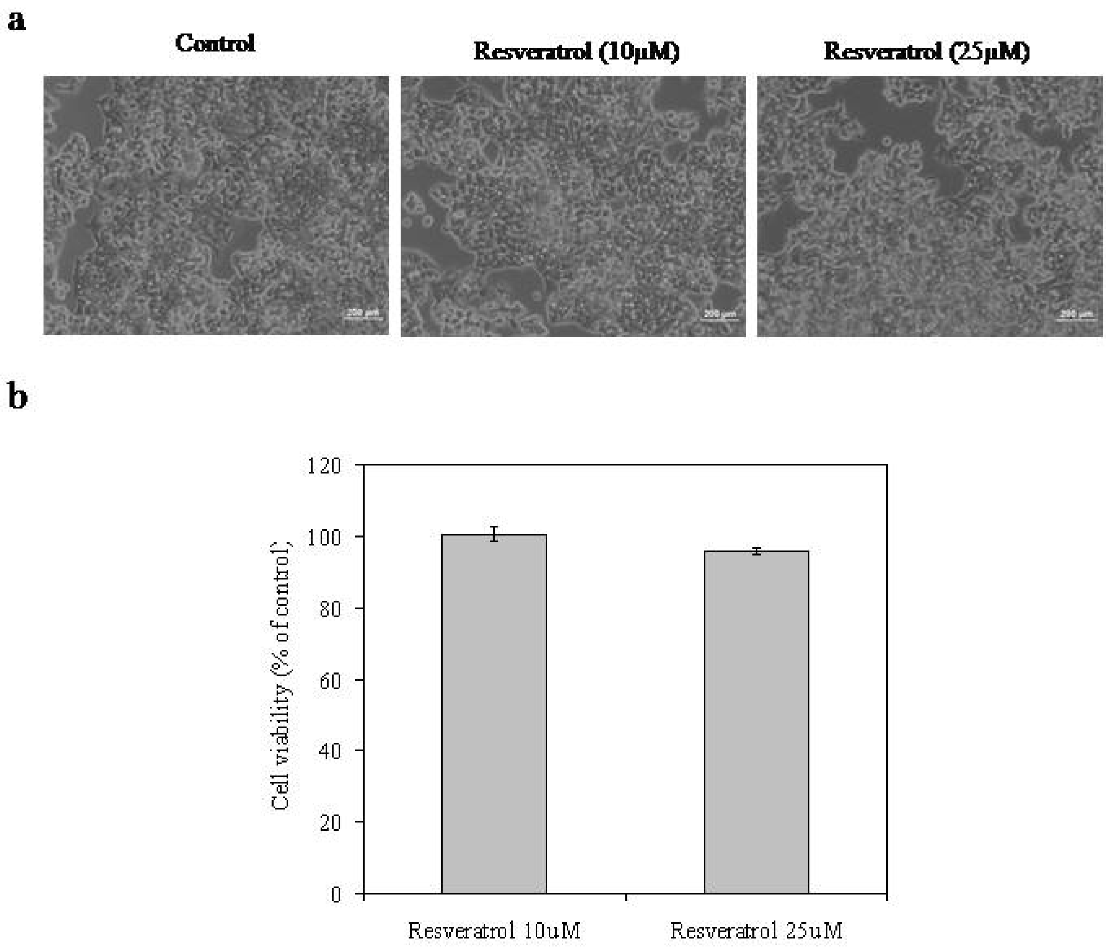

Figure S3. (a) Human CAR (hCAR) and mouse CAR (mCAR) mRNA levels were measured in the cells transfected with hCAR or mCAR expression vectors both increased when compared with the empty vector transfected HepG2 cells. (b) Overexpression of hCAR was further corroborated by western blot of CAR in empty vector and hCAR stably transfected HepG2 cells. (c) CAR siRNA treatment reduced both CAR mRNA and protein levels when compared with siRNA control treated HepG2 cells.

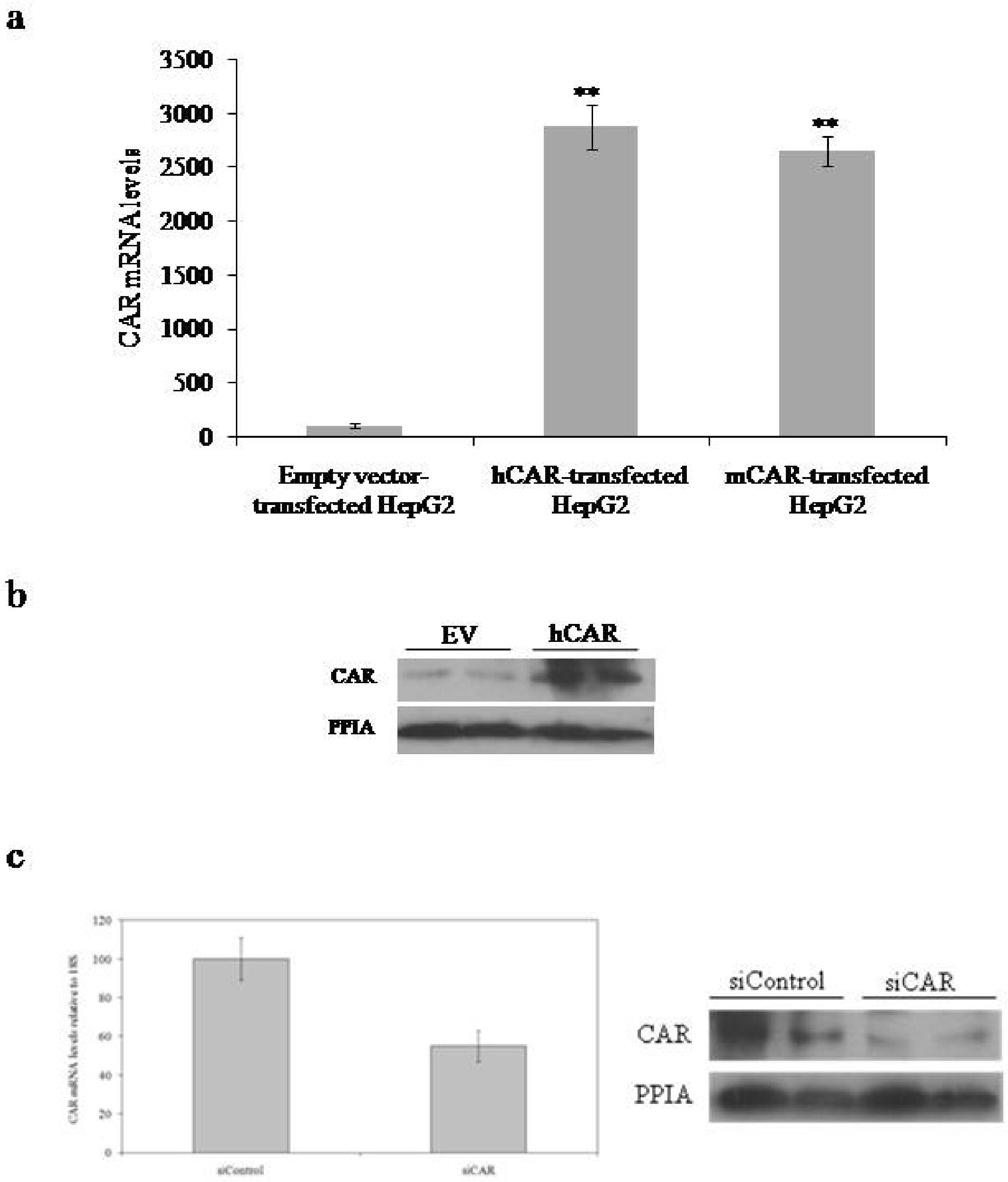

Supplement: Supplementary file 1 — Supplementary Info [file 41598_2017_12509_MOESM1_ESM.pdf]
